# OsDREB2B, an AP2/ERF transcription factor, negatively regulates plant height by conferring GA metabolism in rice

**DOI:** 10.3389/fpls.2022.1007811

**Published:** 2022-10-28

**Authors:** Ziming Ma, Yong-Mei Jin, Tao Wu, Lanjuan Hu, Ying Zhang, Wenzhu Jiang, Xinglin Du

**Affiliations:** ^1^ Jilin Provincial Engineering Laboratory of Plant Genetic Improvement, College of Plant Science, Jilin University, Changchun, China; ^2^ Institute of Agricultural Biotechnology, Jilin Academy of Agricultural Sciences, Changchun, China

**Keywords:** AP2/ERF transcription factor, OsDREB2B, plant height, leaf sheath, gibberellin, rice

## Abstract

The AP2/ERF family is a large group of plant-specific transcription factors that play an important role in many biological processes, such as growth, development, and abiotic stress responses. OsDREB2B, a dehydration responsive factor (DRE/CRT) in the DREB subgroup of the AP2/ERF family, is associated with abiotic stress responses, such as cold, drought, salt, and heat stress, in Arabidopsis or rice. However, its role in regulating plant growth and development in rice is unclear. In this study, we reported a new function of OsDREB2B, which negatively regulates plant height in rice. Compared with wild type (WT), *OsDREB2B-*overexpressing (OE) rice exhibited dwarf phenotypes, such as reduction in plant height, internode length, and seed length, as well as grain yield, while the knockout mutants developed by CRISPR/Cas9 technology exhibited similar phenotypes. Spatial expression analysis revealed that *OsDREB2B* was highly expressed in the leaf sheaths. Under exogenous GA_3_ application, *OsDREB2B* expression was induced, and the length of the second leaf sheath of the *OsDREB2B-*OE lines recovered to that of the WT. OsDREB2B localized to the nucleus of the rice protoplast acted as a transcription activator and upregulated *OsAP2-39* by directly binding to its promoter. *OsDREB2B-*OE lines reduced endogenous bioactive GA levels by downregulating seven GA biosynthesis genes and upregulating eight GA deactivation genes but not GA signaling genes. The yeast two-hybrid assay and bimolecular fluorescence complementation assay showed that OsDREB2B interacted with OsWRKY21. In summary, our study suggests that OsDREB2B plays a negative role in rice growth and development by regulating GA metabolic gene expression, which is mediated by OsAP2-39 and OsWRKY21, thereby reducing GA content and rice plant height.

## Introduction

DREB is a major subgroup of AP2/ERF transcription factor family, which plays an important role in many biological processes such as growth, development and abiotic stress responses in plant. It has been reported that the DREB genes in rice affect abscisic acid (ABA) biosynthesis and signaling, as well as crosstalk between signal transduction pathways for biotic and abiotic stress responses, such as OsDREB2A (Zhang et al., 2013), OsDREB6 (Ke et al., 2014), and OsARAG1 (Zhao et al., 2010). A total of 170 AP2/ERF genes have been identified in rice genome, which are divided into five major groups, namely AP2 (APETALA2), DREB (dehydration-responsive element binding protein), ERF (ethylene responsive factor), RAV (related to ABI3/VP1) and Soloist 5 (Rashid et al., 2012; Gu et al., 2017). Some AP2/ERF transcription factors affect GA metabolism by regulating the expression of GA metabolic genes, thus regulating plant growth and development, such as OsEATB (Qi et al., 2011), OsRPH1 (Ma et al., 2020), and OsAP2-39 (Yaish et al., 2010).

Rice is one of the most important food crops in the world, and it plays an important role in ensuring food security. Plant height is one of the most important factors determining rice plant morphology. An appropriate plant height can reduce the density of leaves, increase yield, and prevent lodging (Sasaki et al., 2002; Spielmeyer et al., 2002). Plant height is regulated by various hormones (Iwamoto et al., 2011), such as gibberellin (GA) (Hedden, 2003; Ueguchi-Tanaka et al., 2005; Liu et al., 2018; Wu et al., 2021), brassinosteroid (BR) (Hong et al., 2002; Tong et al., 2014), indole-3-acetic acid (Aux/IAA) (Huang et al., 2016; Zhang et al., 2016), and strigolactones (SLs) (Zhou et al., 2013). GA can significantly promote elongation of the second leaf sheath in GA-deficient *OsRPH1*-overexpressing rice (Ma et al., 2020). BR can greatly increase the leaf angles of the flag leaves in rice, and BR-insensitive *OsOFP22*-overexpressing rice has a significantly reduced lamina joint angle in flag leaves compared to the WT (Chen et al., 2021). Aux/IAA regulates phototropism/gravitropism, root formation, apical dominance, stem/hypocotyl elongation, and leaf expansion. The overexpression of *OsIAA1* changes the leaf angle (Song et al., 2009), and the overexpression of *OsIAA4* changes the tiller angle (Song and Xu, 2013).

Among these, GA is one of the most important hormones affecting plant height. A total of 136 GA species have been identified; among them, GA_1_, GA_3_, GA_4_, and GA_7_ have been proven to be endogenous bioactive species in plants, and some have at least some intrinsic bio-activities, but they do not exist in vegetative tissues and therefore may have no regulatory function (Hedden, 2002). Numerous rice GA mutants are deficient in the biosynthesis of GA or in the perception of GA signals (Hedden, 2003; Ueguchi-Tanaka et al., 2005; Liu et al., 2018), including GA-deficient and GA-insensitive mutants. GA-deficient mutants are caused by mutations of enzymes involved in GA biosynthesis or GA deactivation, and not in GA perception, as external GA application allows normal height to be recovered (Ashikari et al., 2002; Hedden, 2003; Sakamoto et al., 2004). GA-insensitive mutants are caused by gene mutations involved in GA signal transduction, and the content of endogenous bioactive GAs is usually much higher than that of the WT (Ueguchi-Tanaka et al., 2000; Sasaki et al., 2003; Ueguchi-Tanaka et al., 2005).

In rice, GA metabolic genes include catalytic enzymes in the early steps of GA biosynthesis, such as CPS, KS, KO, and KAO, which are encoded by single genes (Yamaguchi, 2008). In the late stages of GA biosynthesis, GA20ox, GA3ox, and GA2ox are encoded by multi-gene families. Among them, GA20ox and GA3ox convert GA precursors into bioactive GAs through a cascade of catalytic oxidation (Hedden and Thomas, 2012), while GA2ox is critical for GA deactivation (Thomas et al., 1999). In the GA signaling pathway in rice, GID1 encodes a soluble GA receptor (Ueguchi-Tanaka et al., 2005), and GID2 is an F-box subunit of Skp1-Cullin-F box protein (SCF) E3 ubiquitin ligase, facilitating SLENDER RICE 1 (SLR1) degradation by the 26S proteasome in the presence of GA (Sasaki et al., 2003; Itoh et al., 2005).

*OsDREB2B* gene belongs to the DREB subgroup and responds to abiotic stress, such as drought, heat stress, and cold, in Arabidopsis or rice (Chen et al., 2008; Matsukura et al., 2010; Sakata et al., 2014). *OsDREB2B* overexpression effectively improved tolerance to drought stress in rice (Chen et al., 2008) and heat shock and drought stress in Arabidopsis (Matsukura et al., 2010). Interestingly, there is a relationship between cold stress and GA metabolism. For example, under low-temperature conditions, the mRNA expression of *OsDREB2B* is upregulated, while that of the GA biosynthesis genes *GA20ox3* and *GA3ox1* is downregulated in rice (Zhang et al., 2013; Sakata et al., 2014). *SlDREB*, a DREB subgroup gene in tomato (*Solanum lycopersicum*), downregulates the expression of GA-related genes, and its overexpression in transgenic tomato results in dwarfing, including restriction of internode elongation (Li et al., 2012). Overexpression of *OsAP2-39*, an ERF subgroup of AP2/ERF family transcription factor, can reduce plant height in overexpressing transgenic rice by upregulating the GA catabolic gene *OsEUI* (Elongated Uppermost Internode) (Yaish et al., 2010), which encodes for a cytochrome P450 monooxygenase, an enzyme that deactivates GA through an epoxidation reaction (Ma et al., 2006; Zhu et al., 2006; Zhang et al., 2008).

Many AP2/ERF transcription factors regulate plant growth and development and stress response by interacting with other transcription factors, such as BR regulated (Xie et al., 2019), WRKY (Tripathi et al., 2015), MYB (Zeng et al., 2015), and zinc finger transcription factors (Hawku et al., 2021). One of the WRKY transcription factor, OsWRKY21 (LOC_Os01g60640) has been reported to be involved in rice growth and development by regulating the expression of GA metabolism and cell wall biosynthesis-related genes. Overexpressing of OsWRKY21 in rice exhibited a semi-dwarf phenotype, earlier heading dates, and shorter stem internodes (Wei et al., 2021).

In contrast to the wealth of knowledge obtained on stress response, information about the regulation of growth and development by *OsDREB2B* remains limited. In the present study, we focused on *OsDREB2B* regulating plant growth and development. *OsDREB2B-*overexpressing rice possessed a dwarf phenotype of plant height, internode length and seed length in rice. We examined the GA-mediated physiological process of second leaf sheath elongation, the contents of various forms of endogenous GA levels, and the expression alteration of GA-related genes in *OsDREB2B*-overexpressing and knockout mutant. In addition, OsDREB2B downstream target genes and interaction proteins were also elucidated. Our results indicate that OsDREB2B plays a negative role in rice growth and development by regulating GA metabolic gene expression, which is mediated by OsAP2-39 and OsWRKY21, thereby reducing the GA content and plant height in rice.

## Materials and methods

### Plant materials

The full-length CDS of *OsDREB2B* was cloned and fused to the overexpression vector pCUbi1390, which was driven by the maize Ubiquitin promoter by *Pst*I and *Hind*III sites to generate the pUbi::*OsDREB2B* construct. pUbi::*OsDREB2B* was transformed into Japonica rice variety Kitaake *via Agrobacterium* (EHA105-) mediated transformation (Nishimura et al., 2006). The T_3_ homozygous lines were screened by molecular detection and hygromycin resistance selection. The primers used in the plant expression vector construction are listed in **Table S1**.

### Construction and identification of *OsDREB2B* mutants

CRISPR/Cas9 genome editing technology was used to obtain *osdreb2b* mutants. Selection of Cas9/gRNA target site and vector construction were conducted according to previous reports (Li et al., 2017). The OsDREB2B-pBGK032 vector was introduced into *Agrobacterium* EHA105 and transformed into Kitaake (Nishimura et al., 2006). Homozygous *osdreb2b* mutants were screened by testing the specific site of the Cas9 label using PCR analysis and DNA sequencing of the OsDREB2B‐specific editing site. The primer sequences used for plasmid construction and mutant identification are listed in **Table S1**.

### Phenotype analysis, statistical analysis, and growth conditions

All plants were grown in the field at Jilin University in Changchun, Jilin province, China, under normal cultivation condition. The phenotypes of OE and mt lines at seedling stage were evaluated after growing hydroponically for 14 days under 12 h light at 30°C/12 h dark at 24°C. Then, the seedlings were transplanted into the field during the rice-growing season. Seed length, width, and thickness were measured after air drying of fully developed grains. Student’s t-tests were used to determine the statistical significance of phenotypic differences between 10 seedlings of the mt and OE lines.

### GA_3_ treatments

The WT seeds were germinated in distilled water and grown in kimura B culture solution. After culturing for 14 days, the seedlings were treated with 10, 50, and 100 µM GA_3_ (Sigma-Aldrich, Shanghai, China). Leaf sheaths and leaves were collected at 0, 1, 6, and 12 h after treatment. The experiment was performed with three biological replicates.

The exogenous GA_3_ rescue assay was performed as previously described (Ma et al., 2020). WT and transgenic rice seedlings at the three-leaf stage were incubated in kimura B culture solution for 10 days and then treated with 10, 50, and 100 µM GA_3_. The length of the second leaf sheath was measured every 6 h in three trials, with 20 individuals per trial. These experiments were repeated three times. Values are means ± SD.

### Measurement of GA content

The endogenous GA content of the WT, OE, and mt lines was measured by high-performance liquid chromatography-tandem mass spectrometry (HPLC-MS/MS). One gram of shoot tissue from 10d-old rice seedlings was collected for endogenous GA measurements. The quantification of endogenous GAs was performed as described by Li et al. (2017). Three biological replicates and three technical replicates were performed for each sample, and a Student’s t-test was used to determine statistical significance.

### RNA isolation and quantitative real-time PCR analysis

Total RNA was extracted from the leaves of 14d-old rice seedlings using an RNA Prep Pure Plant Kit (Tiangen Co., Beijing, China) and was reverse transcribed using a SuperScript II Kit (TaKaRa, Tokyo, Japan). Real-time PCR was performed using one SGExcel FastSYBR qPCR Mixture (Sangon Biotech, Shanghai, China) on an ABI One Step Plus PCR System (Applied Biosystems, Foster City, CA, USA). Each reaction contained 10 µL SGExcel FastSYBR qPCR Mixture, 0.2 µM primer, and 1 µL template cDNA. The PCR reaction parameters were 95°C for 2 min (1 cycle), 95°C for 15 s, and 60°C for 20 s (40 cycles), followed by melting curve analysis at 95°C for 60 s, 55°C for 30 s, and 95°C for 30 s. The rice *OsUbiquitin* and *OsActin* genes were used as internal controls (Zheng et al., 2015; Mao et al., 2019). Three biological replicates and three technical replicates were performed for each qRT-PCR analysis. The primer sequences used for qRT-PCR are listed in **Table S1**.

### Transactivation activity assay in yeast

The full-length open reading frames (ORF) of *OsDREB2B* were inserted into the pBridge vector to form pBridge-*OsDREB2B.* pBridge-*OsRPH1* was used as a positive control, and the empty pBridge vector was used as a negative control (Ma et al., 2020). The resultant constructs were transformed into yeast strain AH109 (Clontech). The positive transformants were verified on triple dropout media (SD/-Trp-Ade-His) and dropped on quatuor dropout media (SD/-Trp-Leu-Ade-His). All protocols were performed according to the manufacturer’s user manual (Clontech, Mountain View, CA, USA).

### Subcellular localization of *OsDREB2B* in rice protoplasts

For the subcellular localization of OsDREB2B, the coding region of *OsDREB2B* was inserted into the pAN580-GFP vector driven by the CaMV35S promoter to form the OsDREB2B-GFP construct. The D53-RFP fusion protein was used as a nuclear marker (Zhou et al., 2013). Rice protoplast preparation and transformation were performed as previously described by Wang et al. (2016). The transient expression constructs were transformed into rice protoplast cells and incubated in the dark at 28°C for 12–14 h. Fluorescent images were observed using a confocal laser microscope (Carl Zeiss, aJena, Germany).

### cDNA library

A rice cDNA library was constructed using total RNA extracted from 2-week-old rice seedlings. The first-strand cDNA was synthesized by Oligo (dT) primers using the SMARTM cDNA Library Construction Kit (Clontech) with 1 μg of total RNA as template and then double-stranded cDNA was synthesized by long-distance PCR (LD PCR). After a quality check, the dscDNA was inserted into the pGADT7-*Sfi*I vector after *Sfi*I digestion and then transformed into an *E. coli* electro-cell host strain. Six single positive colonies grown on LB medium (Amp 100 µg/mL) were randomly selected to perform PCR amplification with pGADT7 primers: forward, 5’-TAATACGACTCACTATAGG-3’; reverse, 5’-GGCAAAACGATGTATAAATGA-3’. Yeast co-transformed with pGBKT7-53 (+) and pGADT7 served as a positive control, and yeast co-transformed with pGBKT7-Lam (−) and pGADT7 served as a negative control (Chen et al., 2017). The resultant constructs were transformed into yeast strain AH109 (Clontech), and the positive transformants were verified on quatuor dropout media (SD/-Trp-Leu-Ade-His) and dropped on quatuor dropout media (SD/-Trp-Leu-Ade-His+X-gal).

### Yeast-two hybrid assay

The cDNA fragment encoding *OsDREB2B* was amplified and cloned into a pGBKT7 vector to form pGBKT7-*OsDREB2B* as the ‘bait’ using a gateway recombination system. A cDNA library prepared from rice seedlings was used to perform the Y2H screening, and positive clones were identified by DNA sequencing. The coding region of *OsWRKY21* was inserted into the pGADT7 vector as the ‘prey’. The ‘bait’ and ‘prey’ constructs were co-transformed into yeast strain AH109 (Clontech). These positive transformants were verified on triple dropout media (SD/-Trp-Leu-His) and then dropped on quatuor dropout media (SD/-Trp-Leu-Ade-His). All protocols were performed according to the manufacturer’s user manual (Clontech Laboratories).

### Bimolecular fluorescence complementation assays

The cDNA fragment encoding *OsWRKY21* was amplified and cloned into a pXY105 vector carrying the N-terminal half of YFP (nYFP) under the control of a CaMV35S promoter to form nYFP-*OsWRKY21*, and cDNA-encoding *OsDREB2B* was cloned into the pxy 103 vector carrying the C-terminal half of YFP (cYFP) under the control of a CaMV35S promoter to form cYFP-*OsDREB2B*. nYFP-*OsWRKY21* and cYFP-*OsDREB2B* were co-transformed into the rice protoplasts. The D53-RFP fusion protein was used as a nuclear marker. The transformed rice protoplasts were incubated at 23°C in the dark for 12–14 h. Fluorescent images were observed using Axio Observer D1 (Carl Zeiss, Jena, Germany).

### Yeast one-hybrid assays

The full-length CDS of *OsDREB2B* was amplified and cloned into pJG4-5 vector to form pJG4-5-*OsDREB2B*, and the *OsAP2-39* promoter (~1500 bp) was cloned into reporter vector pLacZi-2µ. pJG4-5-*OsDREB2B* and *OsAP2-39pro*:*LacZ* were then co-transformed with the fusion protein into yeast strain EGY48 (Clontech). The transformants were cultured on SD/Trp-Ura-plates at 30°C for 3–4 days. DNA sequencing was used to identify putative positive clones. A single positive colony was streaked on the SD/Trp-Ura chromogenic plate and incubated at 30°C for 3 days (Lin et al., 2007).

### Dual luciferase reporter assay

The *OsAP2-39* promoter (~1500 bp) was inserted into vector pGreen II 0800 containing the luciferase reporter gene (LUC) to generate a reporter vector, and the CDS of *OsDREB2B* was inserted into the Flag vector to generate the effector vector. Subsequently, the reporter vector was co-transformed *via* PEG into rice protoplasts together with an empty Flag vector (negative control) or the effector vector (Flag-*OsDREB2B*) (Yoo et al., 2007). After culturing at 28°C for 12–14 hours, LUC and reniral siferase (REN) activity levels of rice protoplasts were measured using the dual luciferase reporter assay system (E1910, Promega). The transient expression of LUC driven by the *OsAP2-39* promoter was normalized to the expression of the internal control reporter (REN).

## Results

### Tissue-specific and GA-responsive expression profiles of *OsDREB2B*

**Figure 1:**
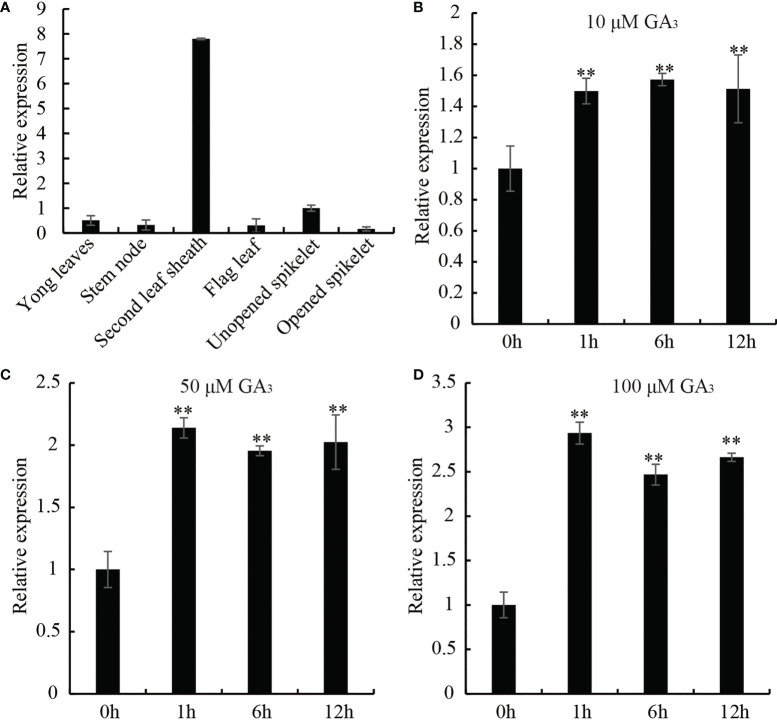
Expression pattern of *OsDREB2B* in rice. **(A)** Transcript levels of *OsDREB2B* in various tissues and organs tested by qRT-PCR. Transcript level of unopened spikelet was set to 1. Data are the mean ± SD (n = 3). **(B–D)** Expression profiles of *OsDREB2B* in response to different concentrations of GA_3_ application analyzed by qRT-PCR. Student’s t-test was used to generate the P values. **Significant differences between the WT and OE plants at P = 0.01.

In order to elucidate whether the biological function of OsDREB2B (LOC_Os05g27930) is related to GA, we examined the tissue-specific and GA-responsive expression of *OsDREB2B* by using qRT-PCR. As shown in **Figure 1A**, the transcript of *OsDREB2B* was expressed in many organs, such as the leaf sheath, young leaf, stem, flag leaf, unopened spike and opened spike. Notably, it was significantly highly expressed in the leaf sheaths, reaching at least eight times the unopened spike, while weak expression was observed in other organs (**Figure 1A**). GA is a hormone that promotes the growth and development of rice, mainly regulating the elongation of leaf sheaths and internodes. To analyze the GA responsive expression of *OsDREB2B*, WT rice treated with different concentrations of exogenous GA_3_, and then its leaf sheaths and leaves were collected at different time points for qRT-PCR. With the increase of GA_3_ concentration, the transcription level of *OsDREB2B* increased: at 100 μM, it rapidly changed 2.9-fold within 1 h (**Figures 1B–D**). Therefore, we suggest that *OsDREB2B* may be involved in GA-regulated biological processes, probably in plant growth and development.

### Overexpressing of *OsDREB2B* in rice has a dwarf phenotype

**Figure 2:**
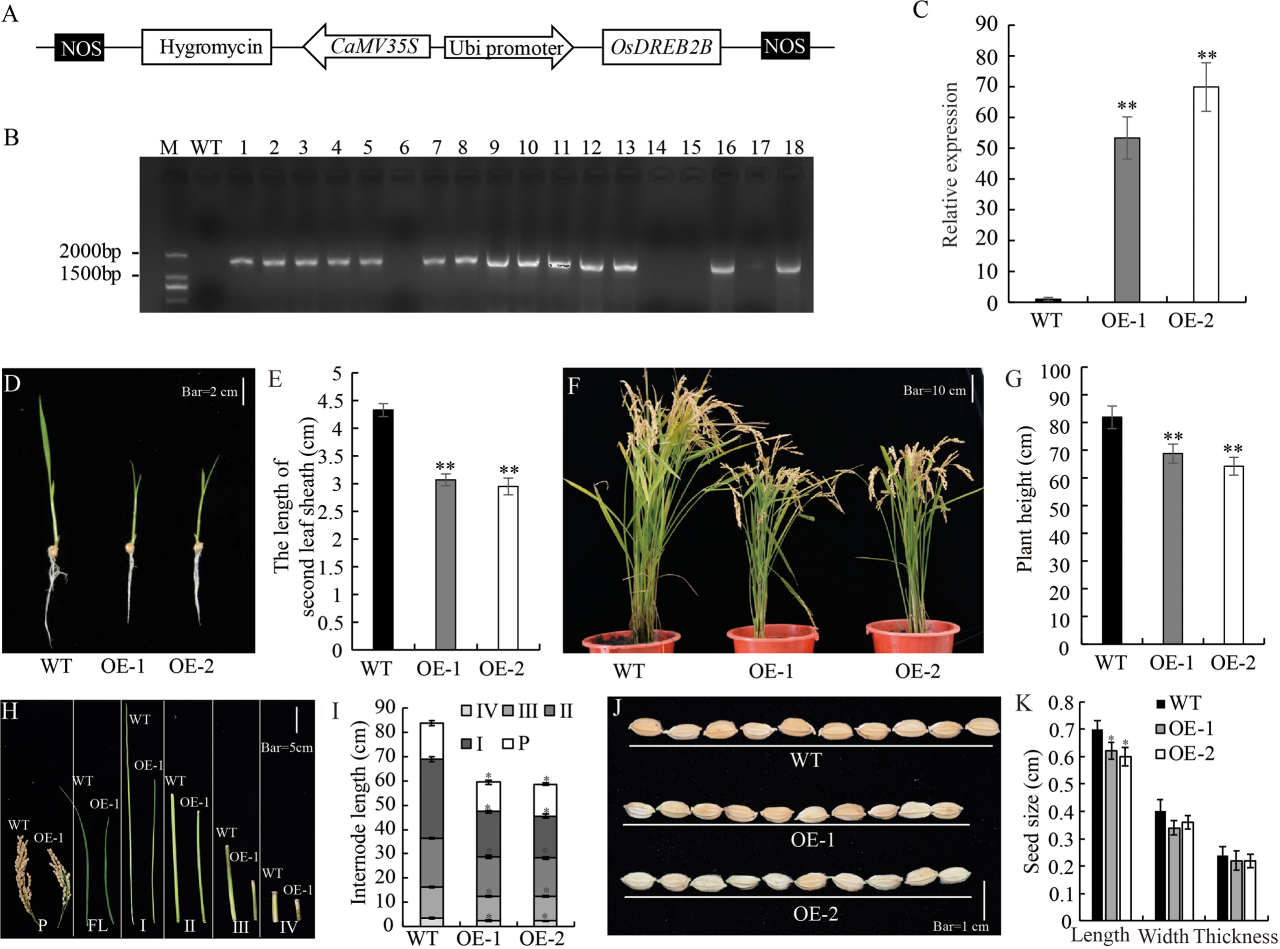
Development and morphology of OsDREB2B-OE rice. **(A)** Schematic representation of *OsDREB2B* plant expression vector. *OsDREB2B* was expressed under the control of maize ubiquitin (Ubi) promoter, and the hygromycin (hgy) gene was under the control of 35S promoter as a selection marker in rice. **(B)** PCR analyses for T_0_ transgenic rice using construct-specific primers. M, marker; WT, Kitaake; 1~18, independent transformants of T_0_ generation. **(C)** The expression of *OsDREB2B* were analyzed by qRT-PCR in WT and DE. The data was presented as the mean ± SE values (n = 3) from two independent experiments. **(D, E)** Whole-plant morphology of the OE lines and the WT at the seedling stage. Bar = 2 cm. **(F, G)** Whole plant morphology of *OsDREB2B*-OE plants and the WT at maturity. Bar = 10 cm. **(H)** Panicles and internodes of the main culms of OE-1 (right) and the WT (left). Bar = 5 cm. **(I)** Internode lengths of OE lines and the WT. **(J)** Seed morphology. Bar = 1 cm. **(K)** Seed size of the OE lines and the WT. Single asterisk indicates significant differences at P ≤ 0.05 compared with the WT and double asterisks indicate significant differences at P ≤ 0.01 compared with the WT in Student’s t-test.

*OsDREB2B* overexpressing and knock-out rice were constructed to elucidate its role in plant growth and development. Plant overexpression vector pUbi::*OsDREB2B* was constructed and transformed into the recipient rice variety ‘Kitaake’ (**Figure 2A**). Through phenotype evaluation, we screened OE lines that exhibited a dwarf phenotype compared to the WT (Kitaake). We obtained 14 independent T_0_ generations of *OsDREB2B-*OE lines, and the expression levels of the transgene were assessed (**Figure 2B**). We selected two lines showing high expression levels of the transgene (OE-1 and OE-2), and their T_3_ homozygous lines were selected for further study (**Figure 2C**). Both OE lines showed dwarf phenotype, with significantly lower plant height, internode length and seed length than WT (**Figures 2D–K**). In addition, many yield component factors, such as panicle number per plant, number of seeds per panicle and seed setting rate, were also significantly reduced (**Table S2**). At the seedling stage, the average lengths of the second sheath of OE-1 (3.07 cm) and OE-2 (2.95 cm) were only 70.9% and 68.1% of the WT (4.33 cm), respectively (**Figures 2D, E**). At maturity, the average plant heights of OE-1 (68.76 cm) and OE-2 (64.16 cm) were only 81.3% and 75.9% of the WT (84.56 cm), respectively (**Figures 2F, G**). A comparison of internode length between the OE lines and the WT showed that four internodes (first, second, third, and fourth) were shorter in the OE lines than in the WT (**Figures 2H, I**). These results suggest that overexpression of *OsDREB2B* has a negative regulatory effect on rice plant height.

**Figure 3:**
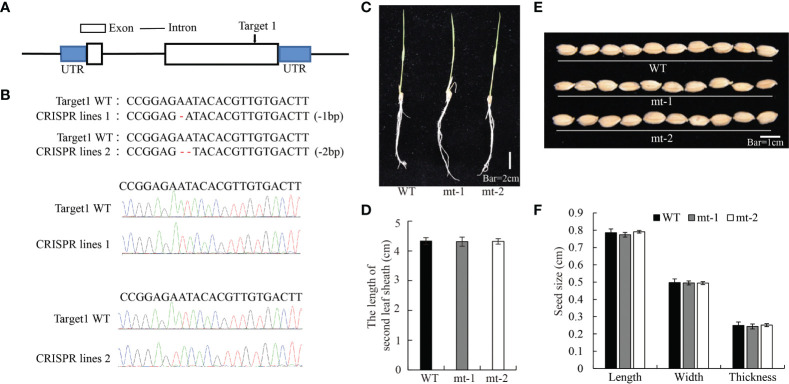
Development and morphology of OsDREB2B-mt rice. **(A, B)** *Osdreb2b* mutants were produced in rice using a cluster of regularly spaced short palindrome repeats (CRISPR)/CRISPR-associated protein 9 (Cas9) system. **(C)** Whole-plant morphology of the mt lines and the WT at the seedling stage. Bar = 2 cm. **(D)** The second leaf sheath length of OE lines and the WT. **(E)** Seed morphology. Bar = 1 cm. **(F)** Seed size of the OE lines and the WT.

To further test the role of OsDREB2B in plant growth regulation, we generated *OsDREB2B-*knockout mutants *(osdreb2b)* using the CRISPR/Cas9 system by *OsDREB2B* genome editing in the WT. By DNA sequencing, two independent knockout lines, mt-1 and mt-2, were selected which were deleted with one or two nucleotides in the second exon of *OsDREB2B*, respectively (**Figures 3A, B**). The length of the second sheath of mt-1 and mt-2 seedlings was similar to that of the WT in 10 d-old seedlings (**Figures 3C, D**). In addition, the length, width, and thickness of the seeds of mt-1 and mt-2 were similar to those of the WT (**Figures 3E, F**). These results suggest that repression of *OsDREB2B* has no effect on rice growth and development.

### Dwarf phenotype of *OsDREB2B-OE* seedlings is rescued by exogenous GA_3_ application in rice

**Figure 4:**
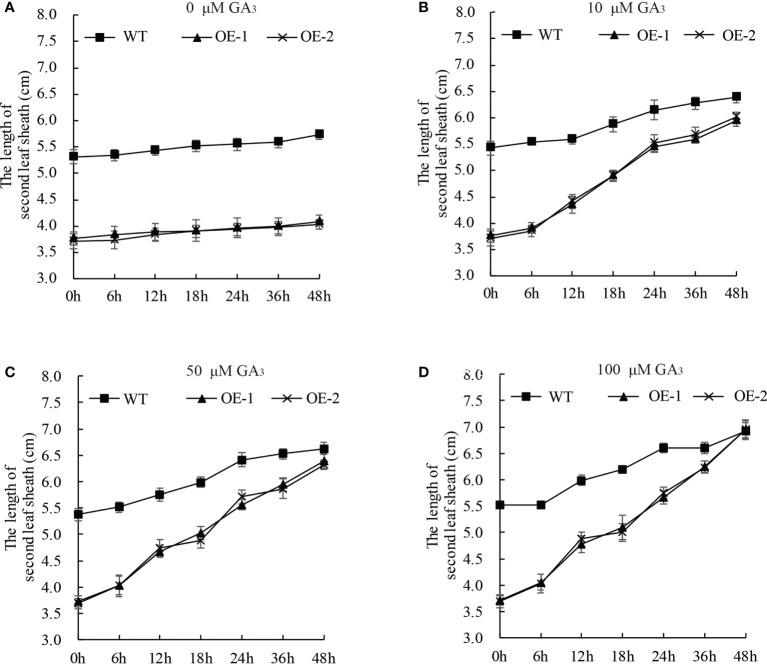
The dwarf phenotype of the OE plants can be rescued by GA_3_. Ten-day-old OE and WT seedlings were treated with 0 **(A)**, 10 **(B)**, 50 **(C)**, and 100 **(D)** μM GA_3_. At least 10 rice seedlings from each individual line were measured. Data are presented as the average of 10 samples per genotype ( ± SD).

GA-deficient rice was dwarfed, and the short length of the second leaf sheath was rescued by exogenous GA_3_ application (Spielmeyer et al., 2002; Ma et al., 2020). To elucidate whether the dwarf phenotype of the *OsDREB2B*-OE lines was due to reduced endogenous GA content, the seedling growth of OE lines and WT rice was investigated under exogenous GA_3_ treatment. 10 d-old seedlings were treated with different concentrations of GA_3_ (0, 10, 50, and 100 μM) at different time points (**Figure 4**). The elongation rate of the second leaf sheath increased with the increase of GA_3_ concentration, and it was faster in the OE lines than in the WT. At the concentration of 100 μM, the length of the second leaf sheath of the OE lines was almost as long as that of the WT plants within 48 h after treatment (**Figure 4D**). In addition, exogenous GA_3_ was also applied in the mt lines, but there was no significant difference with the WT (data not shown). Therefore, exogenous GA_3_ application could completely restore the shortened second leaf sheath of the OE lines, supporting the idea that dwarfism in OE lines was not caused by blocking the GA signaling pathway but might be caused by a deficiency in endogenous GA content.

### Bioactive GA content is reduced in *OsDREB2B-OE* rice

**Figure 5:**
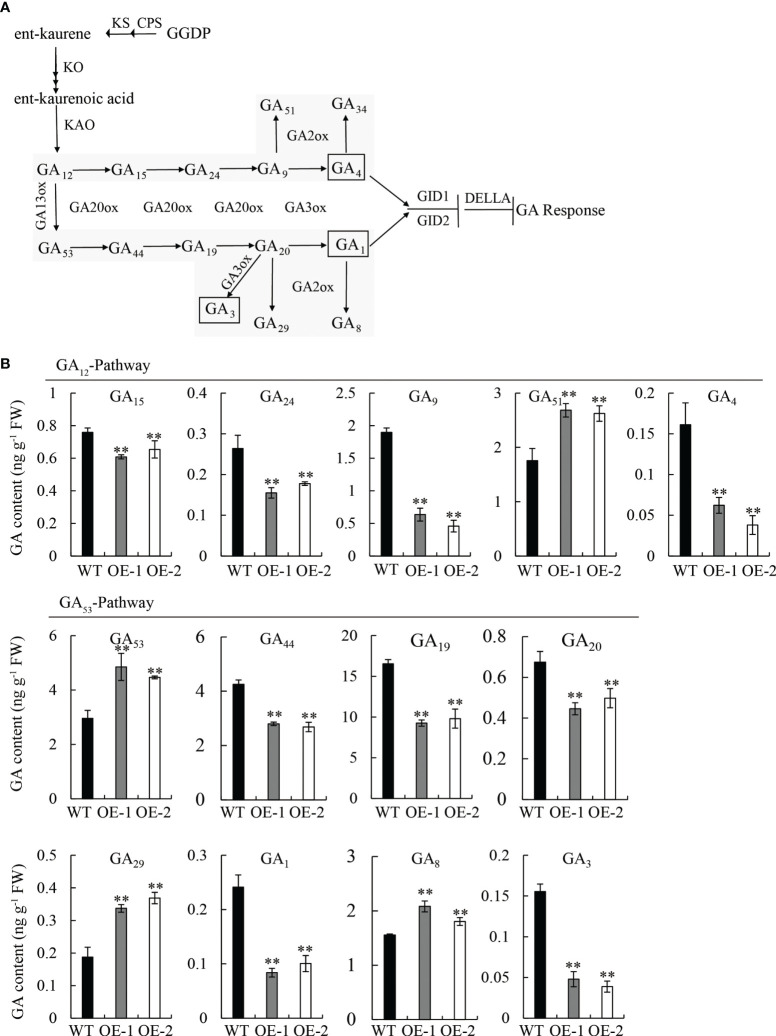
Measurement of endogenous GA in seedlings of the WT and OE lines. **(A)** Comparison of major gibberellin (GA) biosynthetic pathways in plants. **(B)** Content of GA species in the GA_12_-pathway and GA_53_-pathway. FW, fresh weight. Student’s t-tests were used to determine statistical significance. FW, fresh weight; error bars indicate ± SE (n = 3). Double asterisks indicate significant differences at P ≤ 0.01 compared with the WT in Student’s t-test.

To confirm our hypothesis, we quantified the endogenous GA content in 10 d-old seedlings of OE lines and WT by high-performance liquid chromatography-tandem mass spectrometry (HPLC-MS/MS) analysis. The content and distribution patterns of 15 GA species were analyzed, including active forms, as well as their metabolites and precursors (**Figure 5A**). Comparing the GA content between OE and WT: in the GA_12_-pathway, four GA species, including bioactive GA_4_ and its precursors (GA_15_, GA_24_, and GA_9_) decreased, while GA_51_ (the catabolite of GA_9_) increased, and GA_12_ (the first precursor of bioactive GA_4_) and GA_34_ (the catabolite of GA_4_) were not detected; in the GA_53_-pathway, five GA species, including bioactive GA_1_ and GA_3_, as well as their precursors (GA_44_, GA_19_, and GA_20_), decreased; however, GA_53_ (the first precursor of bioactive GA_1_) and two catabolites, GA_29_ and GA_8_, increased (**Figures 5A, B**).

Strikingly, the content of the three bioactive GAs (GA_1_, GA_3_, and GA_4_) was significantly reduced (P ≤ 0.01) in the OE lines compared to the WT, exhibiting a reduction of 62.5, 75, and 68.7%, respectively (**Figure 5B**); however, no significant difference was observed between mutant lines (mt-1 and mt-2) and the WT (**Figure S2**). The overexpression of *OsDREB2B* led to a decrease in endogenous bioactive GA content, but its repression had no effect on GA content, indicating that *OsDREB2B* negatively regulates GA content.

### *OsDREB2B* downregulates GA biosynthesis genes but upregulates GA deactivation genes

**Figure 6:**
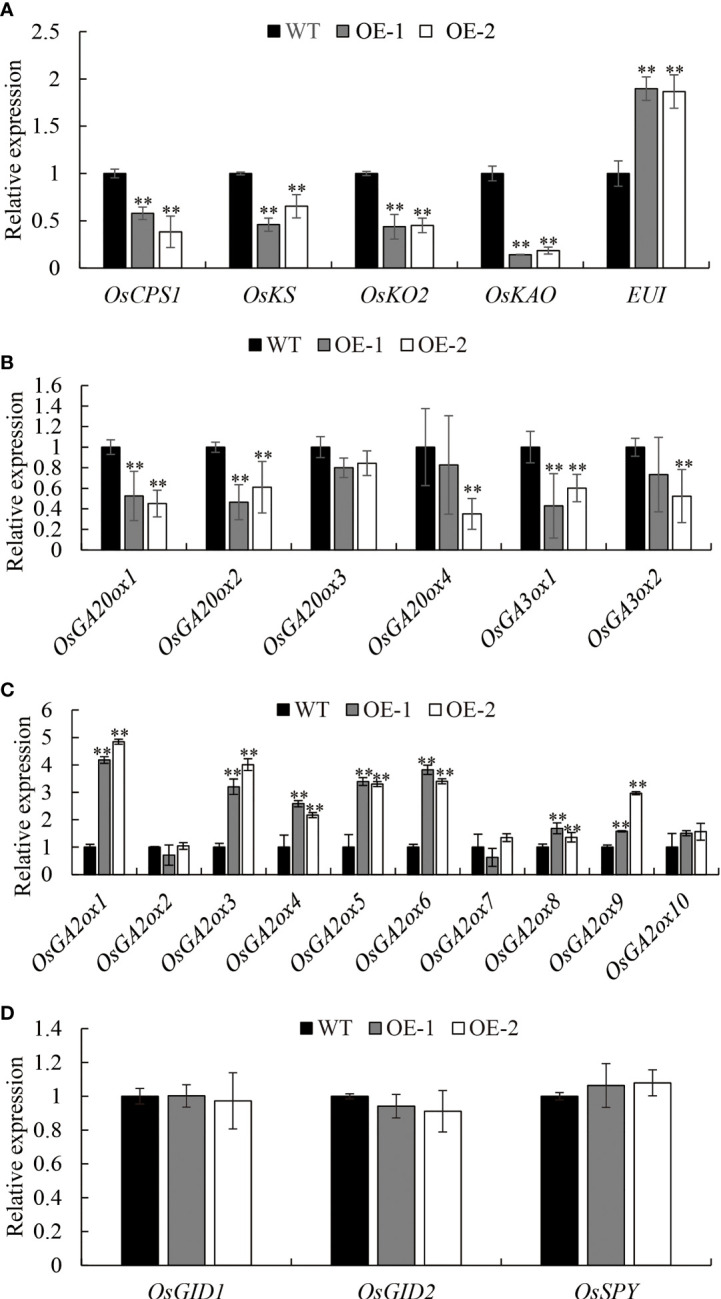
Expression analysis of GA metabolic **(A–C)** and signal genes **(D)**. Transcript levels from the WT were set to 1. Data are the mean ± SD (n = 3). Student’s t-tests were used to generate the P values. **Significant differences between the WT and OE lines at P = 0.01.

To confirm the involvement of *OsDREB2B* as a negative regulator in GA content, the transcription levels of most enzymes that catalyze the early steps and the late steps of GA biosynthesis, as well as GA signaling factors, were examined *via* qRT-PCR in 10 d-old seedlings of OE lines and WT. Interestingly, the expression level of several genes in the OE lines, such as *OsCPS1*, *OsKS*, *OsKO2*, and *OsKAO* in the early GA biosynthesis step, was significantly (P ≤ 0.01) downregulated, which decreased by 44.4, 51.8, 55.5, and 83.7%, respectively, compared to the WT (**Figures 5A**, **6A**). Similarly, the expression level of the late-step GA biosynthesis genes, including *OsGA20ox1*–*OsGA20ox2* and *OsGA3ox1*–*OsGA3ox2*, were significantly (P ≤ 0.01) downregulated in OE lines compared to the WT (**Figures 5A**, **6B**). In contrast, the expression levels of GA deactivation-related genes, such as *OsEUI* (**Figure 6A**), *OsGA2ox1*, *OsGA2ox3-6*, *OsGA2ox8*, and *OsGA2ox9*, were significantly (P ≤ 0.01) upregulated in OE compared to the WT (**Figures 5A**, **6C**).

In addition, the transcript of GA signaling factors *OsGID1*, *OsGID2*, and *OsSPY* in the OE lines did not show significant differential expression compared to the WT (**Figure 6D**), which means that the GA signaling pathway was not blocked by OsDREB2B. This is consistent with the phenomenon of short leaf sheath rescue after exogenous GA_3_ treatment (**Figure 4**). Taken together, *OsDREB2B* negatively regulates bioactive GA content not only by downregulating GA biosynthetic genes, but also by upregulating GA deactivation genes, instead of blocking GA signaling factors.

### *OsDREB2B* functions as a transcriptional activator

**Figure 7:**
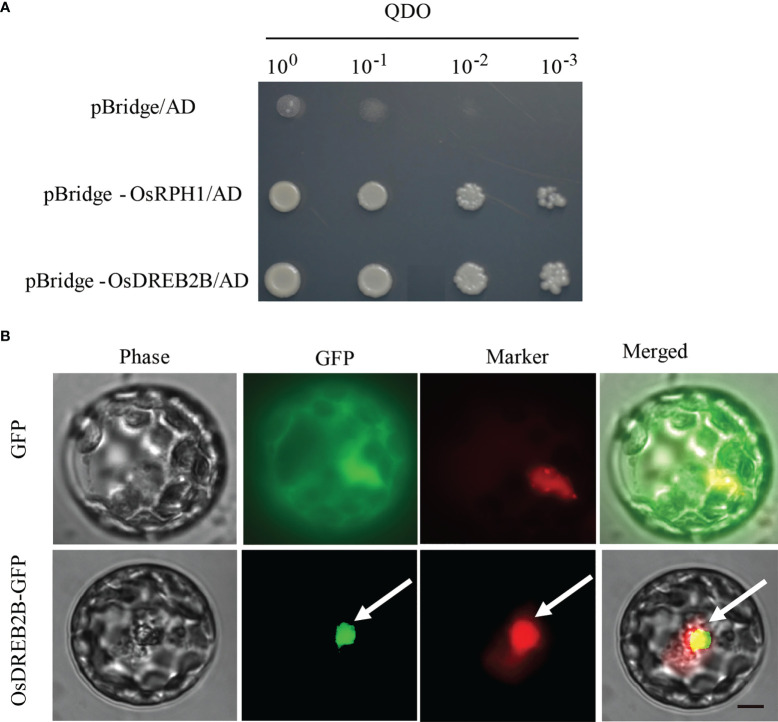
Trans-activation activity and subcellular localization of OsDREB2B. **(A)** Trans-activation analysis of the OsDREB2B protein in yeast. BD, GAL4-DNA binding domain. The OsRPH1 protein was used as a positive control. **(B)** Subcellular localization of OsDREB2B-GFP fusion proteins in rice protoplast cells. D53-RFP was used as a nuclear marker. Bar = 10 µm.

Transcription factors regulate downstream gene expression by binding to *cis*-elements on genomic DNA sequences in the nucleus. To determine whether the OsDREB2B protein has trans-activation activity, it was fused to the GAL4-DNA binding domain in the pBridge vector and then expressed in yeast. The transformants of pBridge-OsDREB2B and the positive control pBridge-OsRPH1 grew well on quatuor dropout media (SD/-Trp-Leu-Ade-His), while the negative control pBridge failed to grow (**Figure 7A**). OsDREB2B fusion with the GAL4 DNA-binding domain of pBridge effectively activated transcription in yeast cells. Compared to the negative control, pBridge-OsDREB2B still grew normally on 75 mM of 3-AT after being transferred to the defective media (SD/-Trp-Leu-Ade-His) containing different concentrations of 3-amino-1, 2, 4-triazole (3-AT) and incubated at 30°C for 3 days (**Figure S3A**). These results indicate that OsDREB2B acts as a transcriptional activator in yeast.

To verify the subcellular localization pattern of OsDREB2B, an OsDREB2B-GFP fusion protein under the control of the CAMV35S promoter (*p35S::OsDREB2B-GFP*) was constructed and then transiently expressed in rice protoplasts. As shown in **Figure 7B**, the control smGFP was uniformly distributed throughout the cell, while the OsDREB2B-GFP fusion protein was exclusively localized to the nucleus. Therefore, we suggest that OsDREB2B functions as a transcriptional activator in rice.

### *OsDREB2B* upregulates the expression of *OsAP2-39* by directly binding to its promoter

**Figure 8:**
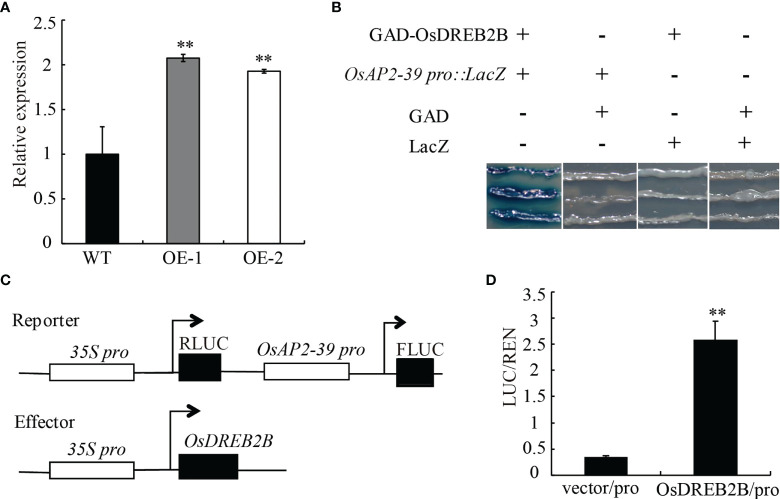
Direct regulation of *OsAP2-39* by OsDREB2B. **(A)** Relative expression of the *OsAP2-39* gene in the WT and OE lines. **(B)** Combination of OsDREB2B and *OsAP2-39* in yeast. GAD-OsDREB2B activates the expression of LacZ reporter genes driven by *OsAP2-39* promoters in yeast. GAD, GAL4 transcriptional activation domain. **(C)** Schematic diagram of the carrier in the dual luciferase reporter system. **(D)** *In vivo* dual luciferase reporter assay. Flag-OsDREB2B and *OsAP2-39pro*:*FLUC*, Flag, and *OsAP2-39pro*:*FLUC* were co-transformed into rice protoplasts and incubated at 28°C for 12–14 (h) FLuc and RLuc activities were measured, and Renilla luciferase (RLuc) was used as an internal control. Student’s t-tests were used to generate the P values. **Significant differences between the WT and OE plants at P = 0.01.

The transcript expression level of *OsAP2-39* was significantly upregulated in the *OsDREB2B-*OE lines compared to the WT (**Figure 8A**). In a promoter assay using PlantCARE (http://bioinformatics.psb.ugent.be/webtools/plantcare/html/), *OsAP2-39* has a DRE/CRT cis-element in the promoter region, however, *OsEUI* does not have any DRE/CRT or GCC-box in the promoter region (**Table S4**). Therefore, we speculated that OsDREB2B directly binds to *OsAP2-39* promoter region to activate its expression, excluding the possibility that *OsEUI* may be a direct downstream target of OsDREB2b.

To confirm the *in vivo* binding of OsDREB2B to the *OsAP2-39* promoter, yeast one-hybrid (Y1H) and dual-luciferase assays were performed. In the Y1H assays, the full-length DREB2B protein was fused to the GAL4 transcriptional activation domain (GAD) and the *OsAP2-39* promoter was fused to the LacZ reporter genes. A blue color reaction occurred when GAD-OsDREB2B and *OsAP2-39_Pro :_ LacZ* were co-transformed into yeast cells (**Figure 8B**). In dual-luciferase assays, the OsAP2-39 promoter was fused to the luciferase (LUC) reporter gene, and *OsDREB2B* was driven by the 35S as the effector (**Figure 8C**). When co-transfected with *OsAP2-39pro*:*FLUC* in rice protoplasts, the relative FLuc/RLuc activity ratios harboring Flag-OsDREB2B were significantly higher than that when co-transfected with an empty vector (**Figure 8D**). These results revealed that OsDREB2B directly binds to the promoter of *OsAP2-39* and activates its expression.

### *OsDREB2B* interacts with *OsWRKY21 in vivo*

Some AP2/ERF transcription factors interact with various proteins to exert redundant functions. To identify the potential interactive protein of OsDREB2B, yeast two-hybrid (Y2H) screening was performed using a rice cDNA library. Autoactivation activity verification of three truncated bait vectors (full length, N-terminal, and C-terminal of OsDREB2B) showed that the C-terminus was required for the autoactivation activity of OsDREB2B. Therefore, the truncated version of OsDREB2B-N without the C-terminus was used for screening (**Figure S3**).

In total, 49 proteins were screened as potential proteins that interacted with OsDREB2B-N (**Table S3**). Most proteins grew normally on the four-deficiency media (SD/-Trp-Leu-Ade-His) and 7 of them had deep blue color reactions (**Figure S4**). Among these, a protein of interest, OsWRKY21 (Serial No. was N-7 in **Table S3** and No.7 in **Figure S4**), which is involved in rice growth and development, was identified.

**Figure 9:**
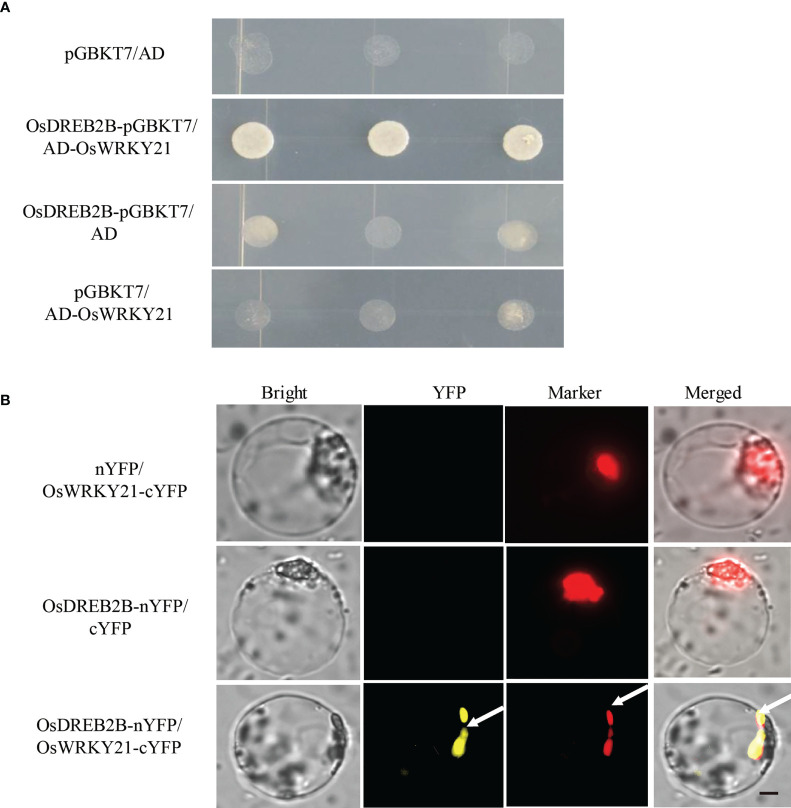
OsDREB2B physically interacts with OsWRKY21 in yeast and plant cells. **(A)** The interaction of OsDREB2B and OsWRKY21 was detected in yeast cells. AD, activation domain; BD, GAL4-DNA binding domain. **(B)** BiFC was used to detect the interaction of OsDREB2B and OsWRKY21 in rice protoplasts. Bright, bright field; YFP, YFP fluorescence; Marker, marker fluorescence. Bar = 10 µm.

To further verify the physical interaction between OsDREB2B and OsWRKY21, a one-on-one Y2H assay was performed. As shown in **Figure 9A**, OsDREB2B-pGBKT7 and AD-OsWRKY21 interacted when co-transformed into yeast. In addition, the interaction between OsDREB2B and OsWRKY21 was verified by the bimolecular fluorescence complementation (BiFC) assay in rice protoplast. Interestingly, the fluorescence signal of YFP was evidently detected in the nucleus of the protoplast when OsDREB2B-nYFP and OsWRKY21-cYFP were co-transfected, whereas no signal was detected in the co-transfection of OsDREB2B-nYFP and cYFP or OsWRKY21-cYFP and nYFP (**Figure 9B**). Therefore, OsDREB2B physically interacted with OsWRKY21 in yeast and in the nucleus of the rice protoplast.

## Discussion

AP2/ERF transcription factor OsDREB2B belongs to the DREB subgroup, which is reported to respond to abiotic stress, such as drought, heat stress, and cold in Arabidopsis or rice (Chen et al., 2008; Matsukura et al., 2010; Sakata et al., 2014). However, little is known about the molecular mechanism of OsDREB2B in plant growth and development in rice. In this study, OsDREB2B acted as a dual regulator of GA metabolic gene expression mediated by OsAP2-39 and OsWRKY21, which reduced GA content and led to the reduction of plant height in rice.

GA is a hormone that promotes the growth and development of rice, mainly regulating the elongation of leaf sheaths and internodes. In our study, the OE lines exhibited dwarf phenotypes, which were mainly manifested in the shortening of leaf sheaths and internode length (**Figures 2**, **S1**), while the phenotypes of the mt lines were similar to the WT (**Figure 3**). These observations revealed that overexpression of *OsDREB2B* disturbs many aspects of plant growth and development, while its inhibition has no effect on the growth of rice.

In addition, exogenous GA_3_ application significantly promoted rice leaf sheath elongation and affected the transcription of OsDREB2B (**Figures 1**, **4**). This phenomenon was similar to that of GA-deficient mutants (Ashikari et al., 2002; Gomi et al., 2004; Sakamoto et al., 2004). Furthermore, the expression of many GA metabolic genes was changed, while that of GA signaling genes showed no difference between the OE lines and the WT (**Figures 5A**, **6**). These results revealed that OsDREB2B is involved in GA metabolism related to plant growth retardation and not the GA signaling pathway.

Among GA metabolic genes, *GA20ox* and *GA3ox* convert GA precursors into bioactive GAs through a cascade of catalytic oxidation reactions (Hedden and Thomas, 2012), while *GA2ox* is recognized as critical for GA deactivation (Thomas et al., 1999). The rice dwarf mutant *d18* and the green-revolution variety, *semi-dwarf 1* (*sd1*), are resulted from mutation in GA biosynthesis genes, *OsGA3ox2* and *GA20ox2*, respectively (Itoh et al., 2001; Spielmeyer et al., 2002). Mutations in *GA20ox* and *GA3ox* in maize (*dwarf1*) and barley (*sdw1/denso*) (Spray et al., 1996; Jia et al., 2015) and overexpression of deactivation genes (*OsGA2ox1*, *OsGA2ox6*, and *OsGA2ox9*) in rice (Sakamoto et al., 2001; Sasaki et al., 2003; Lo et al., 2008; Huang et al., 2010) caused moderate plant height reductions by decreasing the GA content. These circumstances occurred simultaneously in our *OsDREB2B*-OE rice, which is similar to our previously identified AP2/ERF transcription factor, OsRPH1 (Ma et al., 2020). The bioactive GA content in the OE lines was significantly (P ≤ 0.01) lower than that of the WT (**Figure 5**), while that in the mt lines was not (**Figure S2**). Moreover, compared to the WT, most of the bioactive GA precursors were also decreased, while GA_53_ and some inactive catabolites were increased in the OE lines (**Figure 5**). The downregulation of most GA20ox and GA3ox may lead to the accumulation of GA_53_, the first precursor of GA_1_, while the upregulation of GA2ox may lead to the increase of three catabolites, such as GA_29_, GA_8_ and GA_51_ (**Figures 5**, **6**). The altered expression of GA metabolic genes in OE lines agreed with the results of a decrease in bioactive GA content, showing that the GA biosynthesis genes were downregulated and GA deactivation genes were upregulated. These results indicate that OsDREB2B, as a dual regulator of GA metabolic genes, negatively regulates the expression of GA synthetic genes, while it positively regulates GA deactivation genes, resulting in a decrease in bioactive GAs in the rice.

AP2/ERF proteins have been shown to be integrators of biotic and abiotic stress responses by combining with *cis*-acting elements in the promoter region of the target genes, such as ethylene-response elements (GCC-box, core motif:A/GCCGCC) and dehydration-responsive element/C-repeats (DRE/CRT, core motif: G/ACCGAC) (Allen et al., 1998). DREB subgroup proteins contain an AP2-conserved domain that specifically binds to the DRE/CRT *cis*-acting elements of its regulatory gene (Shi et al., 2018). Overexpression of a DREB gene in tomato, *SlDREB*, affects GA biosynthesis by downregulating the key genes involved in GA biosynthesis, thus limiting internode elongation and leading to dwarfing. SlDREB likely acts as a direct repressor of *SlCPS* in plants by binding to the DRE/CRT elements of *SlCPS* (Li et al., 2012). Therefore, we hypothesized that OsDREB2B may have functions similar to those of SlDREB. OsAP2-39 is an AP2/ERF protein belonging to the ERF subgroup that could strongly bind to the GCC box in the promoter of its target GA deactivation gene *OsEUI*, an enzyme that catalyzes 16α, 17-epoxidation of non-13-hydroxylated GAs, which has been shown to deactivate GAs in rice (Yaish et al., 2010). The OsDREB2B protein has transactivation activity and is localized to the nucleus (**Figure 7**). Transcriptional expression of *OsAP2-39* was upregulated in our OE lines (**Figure 8A**), and OsDREB2B was directly combined with the *OsAP2-39* promoter (**Figures 8B–D**). OsDREB2B could be specifically bound to DRE/CRT elements (Chen et al., 2008), and a DRE/CRT *cis*-element was observed in the promoter region of *OsAP2-39* (**Table S4**), indicating that OsDREB2B directly regulates the expression of *OsAP2-39* by binding to a DRE/CRT element in its promoter. In this study, among the genes whose transcriptional expression was altered (**Figure 6**), four GA metabolic genes had at least one GCC box, and five genes had DRE/CRT *cis*-elements in their promoter region (**Table S4**), indicating that OsDREB2B may directly regulate the genes harboring DRE/CRT elements and indirectly regulate genes harboring a GCC-box by binding to the *OsAP2-39* promoter.

The *OsWRKY21-*overexpressing rice showed dwarf phenotypes and regulated the expression of GA metabolism and cell wall biosynthesis-related genes (Wei et al., 2021). In our study, OsDREB2B physically interacted with OsWRKY21, which was verified by the Y2H assay and BiFC assay (**Figure 9**). Interestingly, among the 15 genes whose expression changed, 10 genes contained at least one repeat of a W-box in their promoter (**Table S4**). The OsDREB2B and OsWRKY21 interactions may regulate the expression of the GA metabolic genes that harbor a W-box and/or DRE/CRT *cis*-elements in their promoters. The detailed molecular mechanism of protein–protein interaction on plant growth and development will be elucidated by the generation of *OsDREB2B* overexpression plants in *oswrky21* knockout mutants or/and OsWRKY21 overexpression plants in *osdreb2* knockout mutants in future studies.

**Figure 10:**
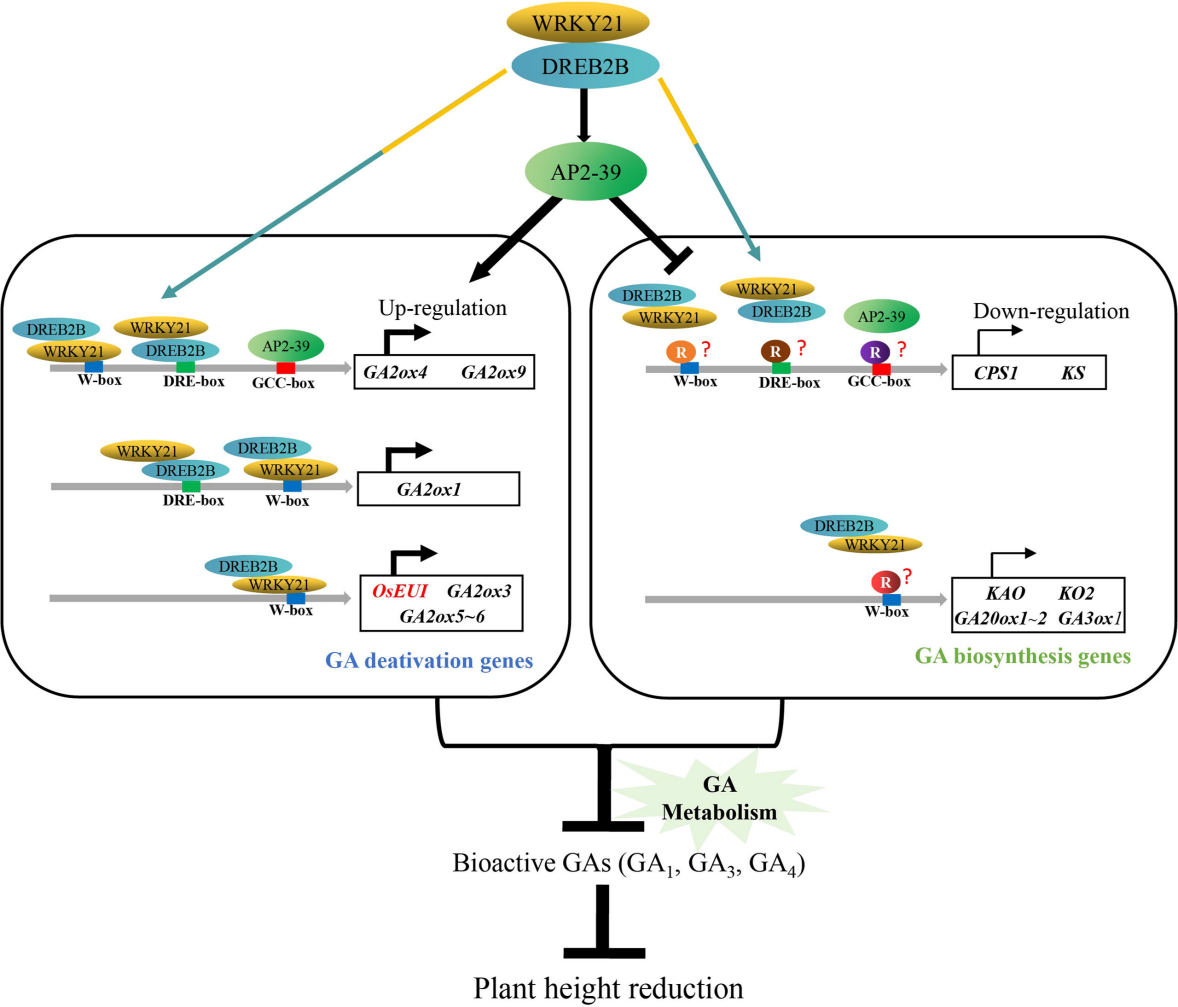
Proposed model of OsDREB2B regulatory mechanism in plant height reduction in rice. Colored spindles indicate different transcription factors, and colored circles indicate different repressors. Colored rectangles indicate the *cis*-element binding sequence. Colored arrows indicate that OsDREB2B/WRKY21 complex can bind directly to the *cis*-element (s) on the promoters of GA metabolic genes. The black arrows represent the activation effect, and the hammer lines indicate the inhibition effect. Curved arrows in the dark indicate the transcriptional expression of genes, and the thickness of the line represents the relative transcription level. Genes in red letters are based on experimental evidence, genes in black letters are based on bioinformatic prediction.

We proposed a model of OsDREB2B controlling plant height in rice through GA metabolism (**Figure 10**). OsDREB2B interacts with OsWRKY21 and directly binds to the promoters, which contain DRE/CRT-box or W-box elements, and indirectly binds to the GCC-box by activating *OsAP2-39* to upregulate GA deactivation genes. In contrast, unidentified transcription repressors inhibit OsAP2-39, and the OsWRKY21/OsDREB2B complex binds to the promoter region of GA biosynthesis genes, resulting in transcription inhibition. The positive and negative regulation of GA metabolic genes leads to a decrease in bioactive GAs, thus reducing plant height in rice.

In summary, OsDREB2B is an AP2/ERF transcription factor that negatively regulates plant height by modulating bioactive GA content *via* downregulating GA biosynthesis genes and upregulating GA deactivation genes by binding to the *OsAP2-39* promoter and interacting with OsWRKY21. The crosstalk mechanism of OsDREB2B on plant growth and stress responses will be further clarified in future studies.

## Data availability statement

The original contributions presented in the study are included in the article/**Supplementary Material**. Further inquiries can be directed to the corresponding authors.

## Author contributions

ZM, Y-MJ, XD, and WJ designed the experiments. ZM, LH, TW, Y-MJ, and YZ performed the experiments. LH and TW analyzed the data. ZM, Y-MJ, TW, XD and WJ participated in the manuscript writing and amending. All authors contributed to the article and approved the submitted version.

## Funding

This research was supported by the Agricultural Major Project of Jilin Provincial Science and Technology Development Plan of China (20210302008NC), Jilin Provincial Agricultural Science and Technology Innovation Project of China (CXGC2021ZY110), and Scientific Research Project of Education Department of Jilin Province of China (JJKH20211130KJ).

## Conflict of interest

The authors declare that the research was conducted in the absence of any commercial or financial relationships that could be construed as a potential conflict of interest.

## Publisher’s note

All claims expressed in this article are solely those of the authors and do not necessarily represent those of their affiliated organizations, or those of the publisher, the editors and the reviewers. Any product that may be evaluated in this article, or claim that may be made by its manufacturer, is not guaranteed or endorsed by the publisher.
