# Supplementary material for: OsDREB2B, an AP2/ERF transcription factor, negatively regulates plant height by conferring GA metabolism in rice
Source: Front Plant Sci. 2022 Oct 28;13:1007811. doi: 10.3389/fpls.2022.1007811 (PMC9650310; doi:10.3389/fpls.2022.1007811)
Supplement: Supplementary file 5 [file DataSheet_1.docx]

**Supplemental Information**

**Figure S1 Panicles and internodes of the main culms of OE-2 and the WT**

**Figure S2 Measurement of endogenous bioactive GAs in WT and Osdreb2b mutants**

**Figure S3Yeast self-activation inhibition assay**

**Figure S4 X-α-gal color reaction in yeast**

**Table S1** Primer sequences used in this study

**Table S2** Agronomic traits between WT and OE plants

**Table S3** List of OsDREB2B interacting proteins by rice cDNA library screening

**Table S4** Promoter analysis of GA metabolic genes and their transcription expression pattern


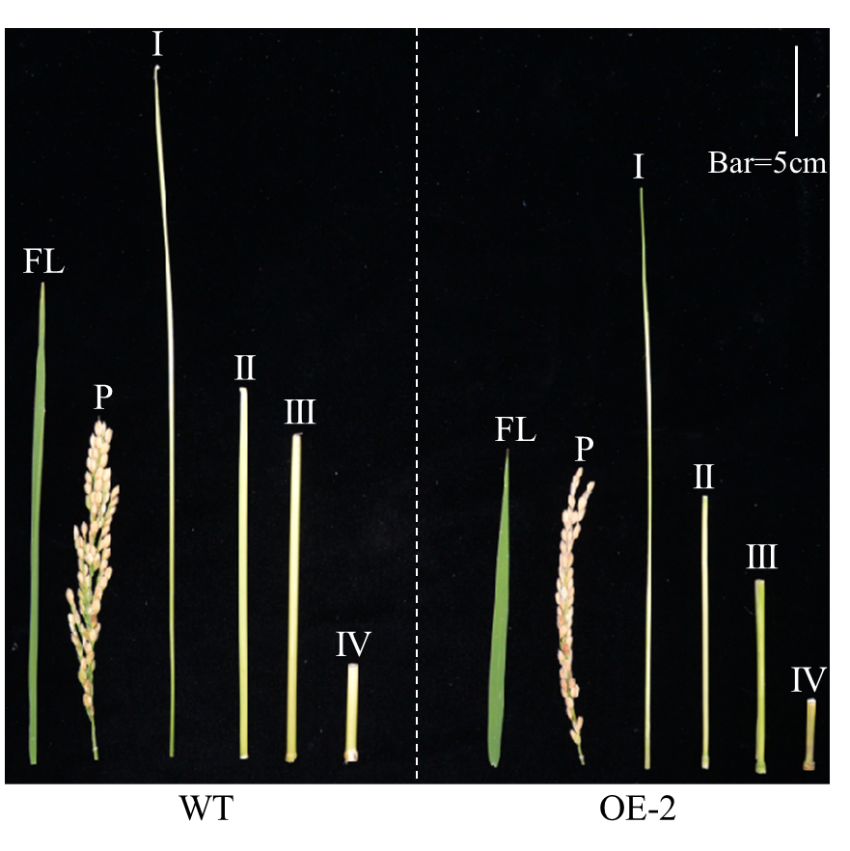


**Figure S1** Panicles and internodes of the main culms of OE-2 and the WT

**
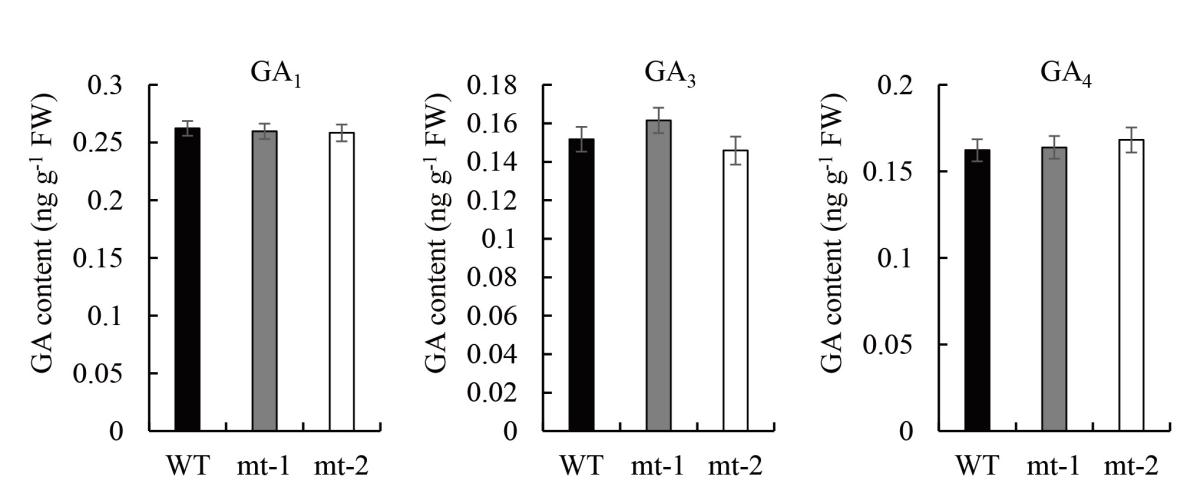
**

**Figure S2** Measurement of endogenous bioactive GAs (GA_1_, GA_3_, GA_4_) in the seedlings of WT and *Osdreb2b* mutants. Three biological replicates and three technical replicates on each sample were performed, and Student’s t tests were used to determine statistical significance. FW, fresh weight; error bars indicate±SE (n=3).

**
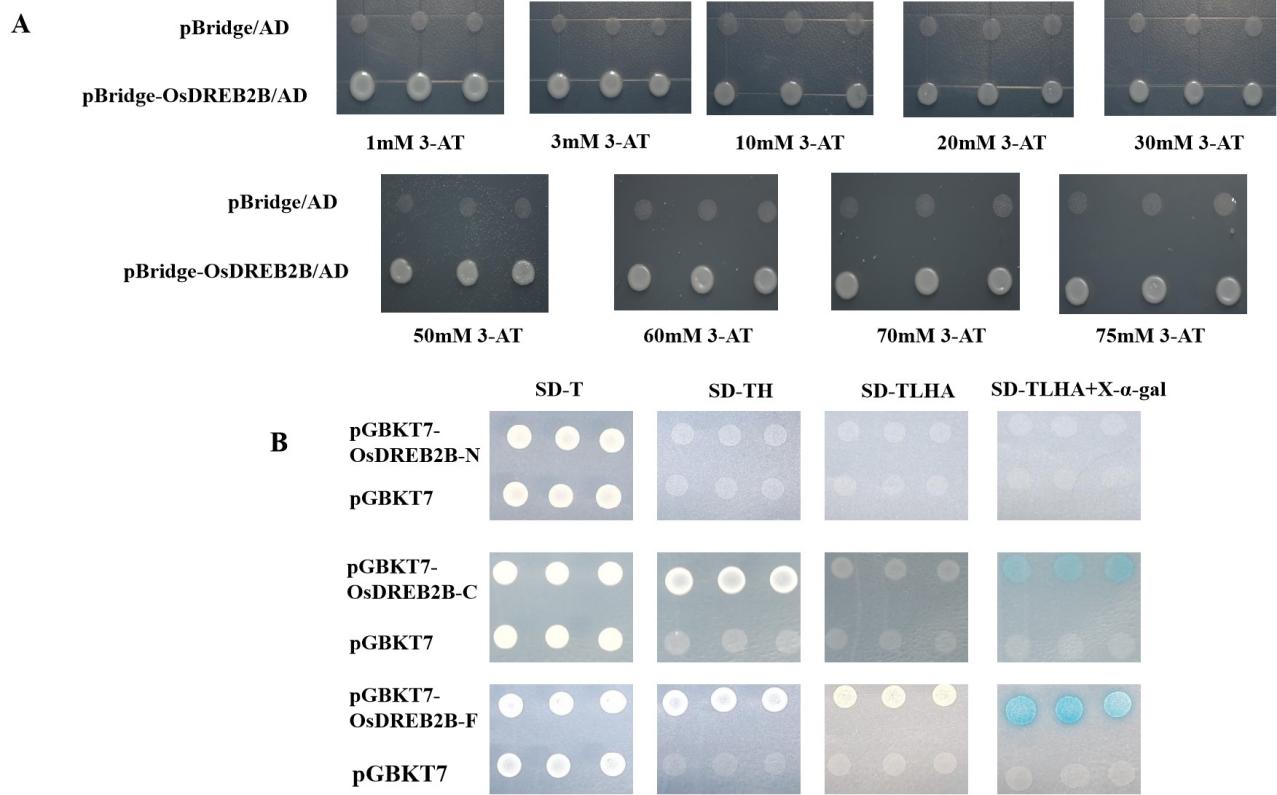
**

**Figure S3** Yeast self-activation inhibition assay. (A) The pBridge-OsDREB2B and pBridge were reconstructed respectively. Yeast one-hybrid analyses of the indicated proteins on minimal synthetic defined media containing SD/-Trp-Leu-Ade-His dropout supplements with different concentrations of 3-AT. (B) Three truncated bait vectors for OsDREB2B, pGBKT7-OsDREB2B-F, pGBKT7-OsDREB2B-N, pGBKT7-OsDREB2B-C indicate truncated constructs of full length, N-terminal and C-terminal of OsDREB2B inserted into pGBKT7, respectively. The indicated proteins were tested on minimal synthetic defined media containing SD/-Trp, SD/-Trp-His, SD/-Trp-Ade-His and SD/--Trp-Leu-Ade-His+X-α-gal dropout.

**
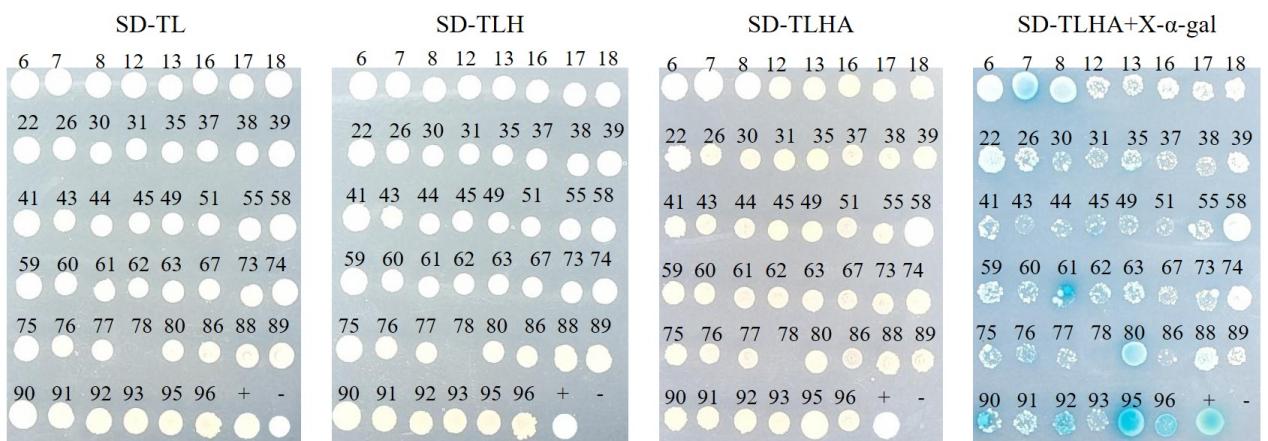
**

**Figure S4** X-α-gal color reaction in yeast. (A) The number was corresponded to the number of Supplementary Table 3, the positive control and negative control corresponding to the + sign and-sign. Yeast cells co-transformed with pGBKT7-53 (+) and pGADT7 were served as a positive control, and co-transformed with pGBKT7-Lam (-) and pGADT7 were served as a negative control. The indicated proteins were incubated on minimal synthetic defined media containing SD/-Trp-Leu, SD/-Trp-Leu-His, SD/--Trp-Leu-Ade-His and SD/--Trp-Leu-Ade-His+X-α-gal dropout.
